# Leadless Pacemaker Implantation in a Patient With Hypoplasia of the Left Brachiocephalic Vein

**DOI:** 10.1016/j.cjco.2022.05.005

**Published:** 2022-05-26

**Authors:** Gabriel Georges, François Philippon, Jean Champagne, Elisabeth Albert, Gilles E. O’Hara

**Affiliations:** a Division of Cardiac Surgery, Quebec Heart and Lung Institute, Quebec, Quebec, Canada; b Division of Electrophysiology, Quebec Heart and Lung Institute, Quebec, Quebec, Canada; c Division of Radiology, Quebec Heart and Lung Institute, Quebec, Quebec, Canada

## Abstract

Venous anomalies are typically asymptomatic and may be discovered unexpectedly at the time of implantation of a cardiac implantable electronic device. We report a case of leadless pacemaker implantation in a patient with hypoplasia of the left brachiocephalic vein who had previously undergone multiple interventions for relapsing right-sided breast cancer. The prevalence and etiology of this anatomic variant remain unknown. However, awareness of its existence may prevent complications during left-sided interventions. such as placement of a central venous line or a cardiac implantable electronic device. Alternative diagnostics and implantation strategies are discussed.

## Résumé

Les anomalies veineuses sont généralement asymptomatiques et peuvent être découvertes de façon inattendue au moment de l’implantation d’un dispositif cardiaque électronique. Nous présentons un cas d’implantation d’un stimulateur cardiaque sans sonde chez une patiente atteinte d’hypoplasie de la veine brachiocéphalique gauche qui avait déjà subi de nombreuses interventions en raison de la rechute d’un cancer du sein du côté droit. On ignore la prévalence et l’étiologie de cette variante anatomique. Toutefois, la sensibilisation à son existence peut permettre de prévenir les complications durant les interventions du côté gauche telles que la pose d’un cathéter veineux central ou l’implantation d’un dispositif cardiaque électronique. Les autres diagnostics et stratégies d’implantation font l’objet de discussions.

Venous anomalies (acquired or congenital) are typically asymptomatic and may be discovered unexpectedly at the time of implantation of a cardiac implantable electronic device. Rarely, venous anomalies result from chemotherapy or radiotherapy. We report a case of leadless pacemaker implantation in a patient with a severely hypoplastic left brachiocephalic vein who had previously undergone multiple cycles of radiotherapy for relapsing right-sided breast cancer.

**Figure 1:**
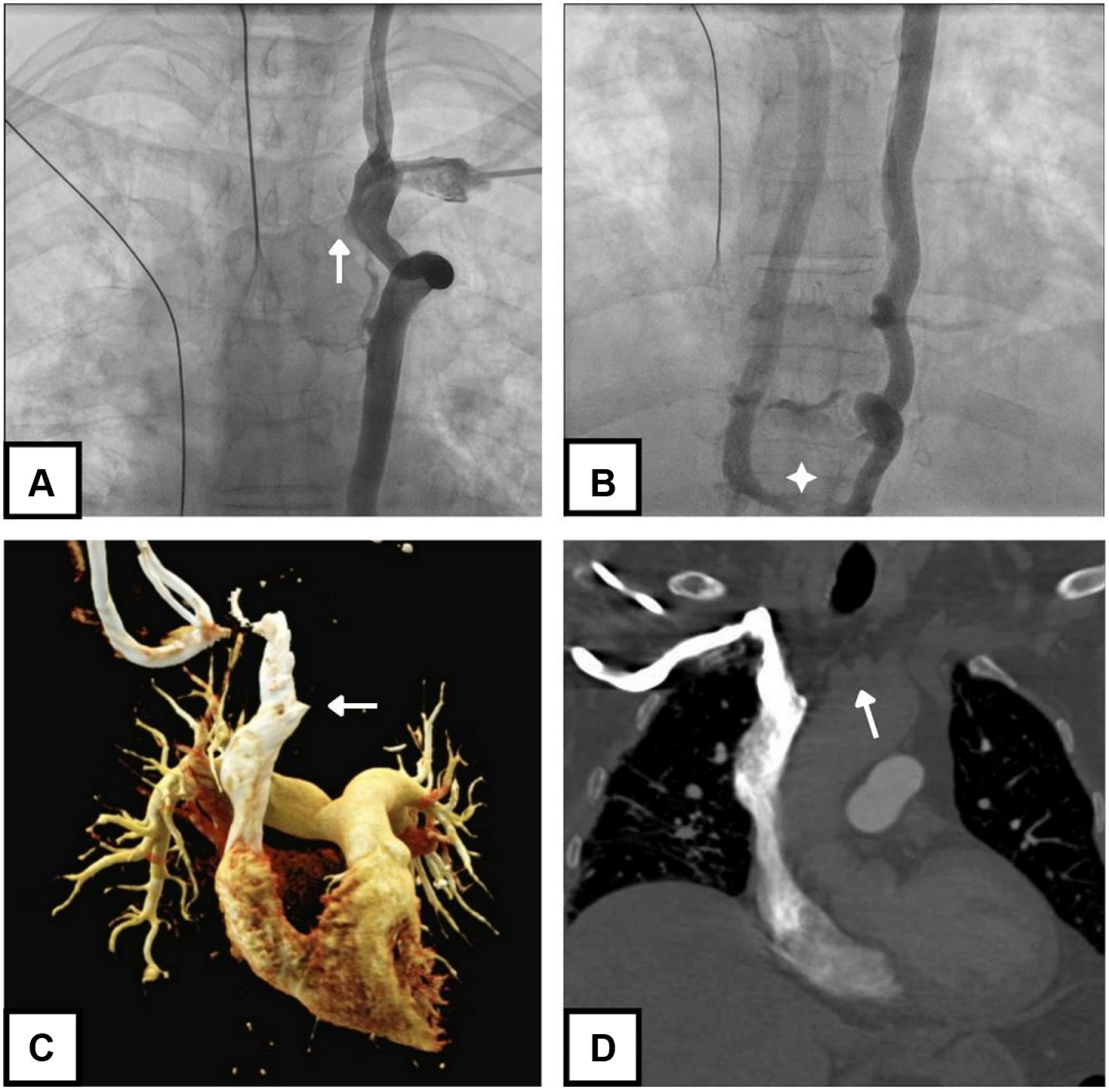
(**A, B)** Intraoperative left subclavian venogram showing drainage of the left upper body into the left hemi-azygos. (**A**) The **arrow** points to a distal brachiocephalic vein bud. (**B, star**) The hemi-azygos vein communicates with the azygos vein via a large collateral at the level of T10. (**C, D**) Contrast-enhanced computed tomography reconstruction after right-arm contrast injection revealing (**C, arrow**) a proximal brachiocephalic vein bud. The left jugular and subclavian veins drain into the hemi-azygos vein. The brachiocephalic vein is severely hypoplastic and is difficult to visualize with a right-sided contrast injection. (**D, arrow**) The expected localization of the brachiocephalic vein is illustrated.

A 78-year-old woman with symptomatic transient third-degree atrioventricular block was referred for dual-chamber pacemaker implantation. The patient had a history of 2 right-sided breast cancer relapses, for which she underwent repeated surgeries and radiation therapy cycles between 1983 and 1993. A venogram at the time of implantation showed the absence of a left brachiocephalic vein, with drainage of the left jugular and subclavian veins into a left hemi-azygous vein (Fig. 1, panel A, B). Based on the patient’s history of repeated right-sided breast cancer surgery, a right-sided pacemaker implantation was not considered. Contrast-enhanced computerized tomography revealed a patent severely hypoplastic brachiocephalic vein (Fig. 1, panel C, D). An aberrant left subclavian artery was fortuitously discovered, running behind the esophagus. No anomalies were found at the level of the inferior vena cava and femoral veins. The patient underwent implantation of an atrioventricular-synchronous leadless pacemaker (Micra AV, Medtronic, Minneapolis, MN) via the right femoral vein on the same day and was discharged the next morning.

The prevalence of this anatomic variant remains unknown, and the etiology of an absent or hypoplastic brachiocephalic vein remains speculative.^1^^,^^2^ However, acknowledgment of its existence may prevent complications during left-sided interventions, such as placement of a central venous line or cardiac implantable electronic device. Interestingly, our patient was also found to have an aberrant left subclavian artery. The 2 anomalies, however, do not share common embryologic structures. Although the left subclavian artery derives from the left seventh cervical intersegmental artery, the left brachiocephalic vein develops during the anastomosis of the right and left anterior cardinal veins.^2^^,^^3^ Given that the major tributaries of the superior and inferior vena cava derive from distinct embryologic veins, the discovery of an anomaly in either system should not discourage accessing of the other. In our patient, no anomalies were found at the level of the inferior vena cava tributaries, and the femoral vein access route was utilized without any complications. Alternative diagnoses to brachiocephalic vein hypoplasia include brachiocephalic vein stenosis secondary to central venous chemotherapy catheters or radiation therapy. Yet, our patient had refused chemotherapy, and although radiation-induced arteriopathy is well documented, venous injury is less common. Only a handful of radiation-induced superior vena cava or brachiocephalic vein occlusion cases have been reported previously. All cases followed high-dose mediastinal or cervical radiation.^4^ Moreover, the absence of collaterals in our patient disputes an acquired etiology.

We report a rare case of brachiocephalic vein hypoplasia discovered at the time of left-sided pacemaker implantation. Although this anatomic variation is rare, knowledge of its existence may allow avoidance of vascular complications. In this patient with a history of repeated right breast cancer surgery, alternatives included implantation of a leadless pacemaker, a transvenous femoral pacemaker, or epicardial leads. Investigation of the lower-body venous drainage anatomy revealed no further anomalies, and a leadless pacemaker was implanted.

**Novel Teaching Points**

• An absent or hypoplastic left brachiocephalic vein is rare, but this possibility should be kept in mind when performing left-sided interventions.
• The major tributaries of the superior and inferior vena cava derive from distinct embryologic veins.
• Venous anomalies are most often solitary. When a venous anomaly is discovered, alternative transvenous implantation strategies should be considered.
• Leadless pacemaker implantation can be successfully performed in patients with abnormal upper-body venous drainage as an alternative to surgical epicardial leads.
